# Epididymis rhabdomyoma: A case report and literature review

**DOI:** 10.1186/1746-1596-7-47

**Published:** 2012-04-20

**Authors:** Yang Han, Xue-shan Qiu, Qing-chang Li, Yu-chen Han, Xu-yong Lin, Qing-fu Zhang, Jian Wang, En-hua Wang, Ze-liang Li

**Affiliations:** 1 Department of Pathology, College of Basic Medical Sciences and First Affiliated Hospital of China Medical University, Shenyang, China; 2 Department of Urology, First Affiliated Hospital of China Medical University, Shenyang, China

**Keywords:** Rhabdomyoma, Epididymis, Epididymis rhabdomyoma immunohistochemistry

## Abstract

Genital rhabdomyoma is very rare tumor that usually occurs in the vulvar of young women. Epididymis rhabdomyoma in a young man is extremely uncommon and has rarely been reported. Here, we report a case of epididymis rhabdomyoma of a 17-year-old man and review the literatures.

**Virtual slide:**

The virtual slide(s) for this article can be found here: http://www.diagnosticpathology.diagnomx.eu/vs/1177628224692794

## Background

Rhabdomyoma is an exceedingly rare benign tumor of striated muscle. It can be divided into the following categories: cardiac rhabdomyomas and extracardiac rhabdomyomas, which are relatively rare [1]. Genital rhabdomyoma is even more uncommon, usually occurs in the vulva of young women [2,3]. Epididymal rhabdomyoma is extremely rare in the world and has rarely been reported [4,5]. Here, we report a case of epididymis rhabdomyoma of a 17-year-old man and review of the literatures.

## Clinical history

**Figure 1:**
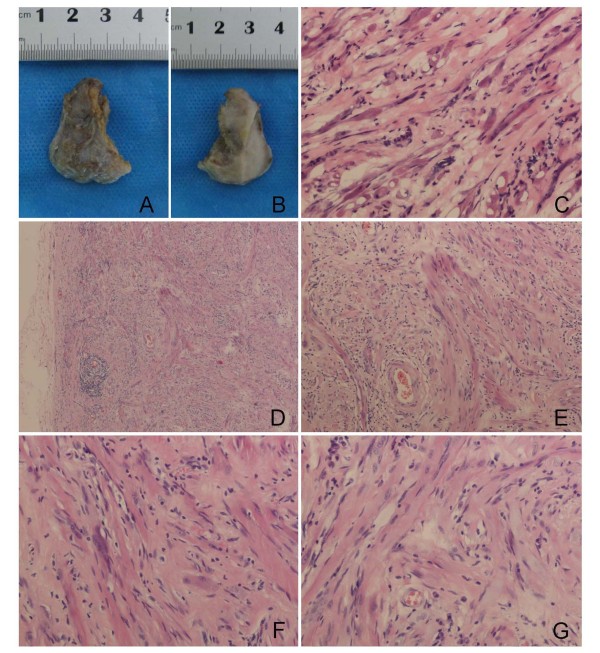
**A and B, gross appearance of tumor**. The mass with part of thin fibrous capsule (**A**) and the cut surface was solid, tan-white and had a faint fascicular pattern (**B**). **C**, The frozen section, a little ice crystals in the tissue, HE400. **D-G**, Bland-appearing, round, spindled, elongated, ribbon cells with abundant eosinophilic cytoplasm and eccentrically placed, oval nuclei rhabdomyocytes with cross-striations. No mitosis or atypia is noted. Mononuclear cells infiltrate. **D**, HE100; **E**, HE200; and **F&G**, HE400.

A 17-year-old Chinese man presented with a painless, indurated, right testicular mass and no other associated symptoms. The patient felt the mass increased slowly during the past 2years. He denied any history of trauma to testes, systemic TB (tuberculosis), genital infections, or STD (sexually transmitted diseases). The patient denied his family history ever had male genital tumor, such as von Hippel-Lindau disease (a risk factor of epididymal papillary cystadenomas) or other genitourinary diseases [6]. During the past 2years, he tried a variety of non-surgical treatments, including traditional Chinese medicine, but the mass had no significant change. At first, his mother, a farmer, believed sexual intercourse might reduce the mass, but one year after married, the mass didnt shrink. Physical examination showed a firm, nontender mass in the right scrotal sac, measured about 1.5cm1.0cm1.0cm and was considered originated from epididymis. The left scrotal sac was unremarkable. The penis was uncircumcised. The rectal examination was within normal limits. Urinalysis revealed no significant abnormalities. Serum human chorionic gonadotropin (hCG) and a-fetoprotein (AFP) levels were not elevated. Ultrasound revealed a slightly inhomogeneous nodule in the head region of the right epididymis, about 1.51cm1.13cm. Inguinal exploration with possible right radical orchiectomy versus right epididymis tumor resection was scheduled. Surgical findings: A 1.5cm1.0cm1.0cm firm mass involved the head of the right epididymis. There was no obvious abnormal in the right testis and the tunica vaginalis was intact. Intraoperative frozen section diagnosis of the epididymal mass considered a benign tumor, possibly leiomyoma (Figure1, C). Conservative excision of the mass with preservation of the right epididymis and testis was performed. No adjuvant treatment was performed and the patient is well, without recurrence 6months after surgery.

## Pathology

### GROSS

A firm mass with tan white fibrous capsule measured 1.5cm1.0cm1.0cm in the head of epidymis (Figure1, A). The cut surface of the tumor was solid, tan-white, had a faint fascicular pattern (Figure1, B). No hemorrhage or necrosis was noted.

### Histology and immunohistochemistry

The tumor was fixed in 10% formalin and embedded in paraffin. Several 4-m sections were cut from each paraffin block. Hematoxylin-eosin (HE) and immunohistochemical (IHC) stains were performed. IHC staining was performed using the streptavidin-peroxidase system (Ultrasensitive; MaiXin Inc., Fuzhou, China) according to the manufacturer's instruction. Commercially available prediluted monoclonal antibodies against the following antigens were employed: Actin (sm, smooth muscle) (1:200; Mouse mAb (DE-B-5), Merck), CK (1:200; Mouse mAb (B311.1), Merck), S-100 (1:200; Mouse mAb (1B2), Merck), CD34 (1:200; Mouse mAb (QBEnd/10), Merck), Vimentin (1:200; Mouse mAb (V-9), Merck), Desmin (1:200; Mouse mAb (DE-B-5), Merck) and Ki-67 (1:200; MIB1, Dako). The immune reactions were visualized with the use of DAB as the chromogen (Sigma-Aldrich Co, St Louis, Mo, USA). All internal and external controls worked appropriately.

## Results

**Figure 2:**
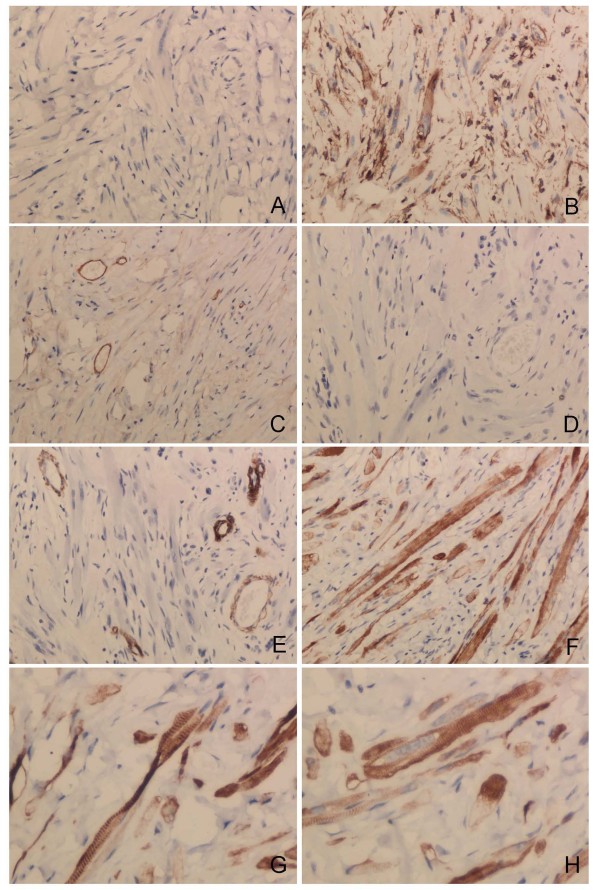
**IHC staining of the tumor with strong staining of tumor cells for Vimentin (B, 200) and Desmin (F, 200; G&H, 400)**. Negative for CK (**A**, 200), CD34 (**C**, 200), S-100 (**D**, 200) and Actin (sm) (**E**, 200). Note the striate in the tumor cells (**G&H**), CD34, staining of vascular endothelial cells (**C**) and actin (sm), staining of vascular myoepithelial cells (**E**).

Microscopically, the mass showed a proliferation of rhabdomyocytes in a background of dense connective tissue. Microscopic examination found multiple defined nests of mature, round to fusiform,elongated, ribbon cells with abundant eosinophilic cytoplasm and eccentrically placed, oval nuclei. The nuclei were uniform and round. No pleomorphism, atypia, mitotic figures or pathological mitotic were identified. Focal adipocyte collections were noted among the dense fibrous connective and little mucilage tissue. A scant, patchy mononuclear cell infiltrate, perhaps due to slight chronic inflammation caused by tumor growth, was present throughout the tumor (Figure1, D-G). Immunohistochemical stains showed these lesional cells to be negative for CK, CD34, S-100, Actin (sm), and positive for Vimentin and Desmin. Ki-67, a proliferation marker, was positive in less than 1% of lesional cells (Figure2). The final diagnosis was right epididymis fetal rhabdomyoma.

## Discusson

Rhabdomyomas are benign tumors of striated muscle cells and generally divided into the following categories: cardiac rhabdomyomas, which are relatively common, and extracardiac rhabdomyomas (occurring outside of the heart), which are rare (comprising only 2% of all tumors with striated muscle differentiation) [2,3]. Based on clinical manifestations and morphological characteristics, the extracardiac forms of rhabdomyoma are subclassified into 4 distinct types: (1) the fetal type, a rare form that affects the head and neck region but occurs in both children and adults. Within the fetal subtype, there is a wide spectrum of histology reflecting the degree of differentiation [7,8]; (2) the adult type, usually found in the head and neck region of the older person and slowly growing, more than 40% recur. This type tumors can be multinodular, cells are rounder, and there are crystalline cytoplasmic inclusions of hypertrophic Z-band material [9-11]; (3) the genital type, a tumor-like polypoid or cystic mass that has been described commonly in the genital tract of middle-aged women with a mean age of 42years. This type resembled fetal rhabdomyomas in architecture but showed greater maturity of the myocytes [4,12,13]; and (4) rhabdomyomatous mesenchymal hamartomas, a peculiar striated muscle proliferation that occurs chiefly in the periorbital and perianal region of infants and young children. The pathologic subtype does not necessarily reflect the age of the patient.

The differential diagnosis of an epididymal tumor includes TB, spermatic granuloma, adenomatoid tumor (a common tumor in this location), mesothelioma, papillary cystadenoma (especially if the patient has a family history of von Hippel-Lindau disease), and embryonal rhabdomyosarcoma. The right epididymal tumor removed from the young patient showed clinicopathologic features typical of the genital variant of extracardiac rhabdomyoma. Additional histologic considerations consist of other benign processes, such as leiomyoma and fibromatosis; the presence of cross-striations and IHC steins rule out leiomyoma or fibromatosis. Histologically, the fetal variant of rhabdomyoma shares many features of the genital variant, and usually has a more myxoid and less collagenous stroma with proliferation of immature mesenchymal cells and is more cellular. Embryonal rhabdomyosarcoma should be considered in the differential diagnosis, in which one would expect to find necrosis, increased mitotic activity, abnormal mitoses, nuclear atypia, pleomorphism, anaplasia and a high proliferation index, e.g. Ki67>5% [14].

Here, we report this quite rare tumor and provide comprehensive figures, including gross appearance, HE and IHC staining. This is the third epididymal rhabdomyoma described in the English literature [4,5]. The first two cases are both 20years old. They did not present specific symptoms, and a tumor mass in left epididymis (5.5cm4.0cm2.5cm and 5.0cm4.0cm2.0cm respectively) was the only finding. The probable origin is the cremaster muscle. Additionally, there are other six rhabdomyomas in the male genitourinary tract described in English, two in the spermatic cord (67-year-old man, 3.0cm2.0cm2.0cm,17-year-old man, 3.5cm2.4cm2.1cm, respectively) [10,15], one in the tunica vaginalis (19-year-old man) [12], one in prostate (nineteen-year-old white man) [16] and two paratesticular (10months of infant, 1.5cm and 55-year-old man, 6.0cm5.5cm4.5cm, respectively) [17,18]. When faced with a pleomorphic mesenchymal tumor in the male genitourinary tract, pathologists should remember the diagnosis of rhabdomyoma. To our knowledge, rhabdomyomas exhibited benign behavior and low recurrence rate in other sites. Further documentation and follow-up of such cases will help better define the biological behavior and prognosis of the rhabdomyomas.

## Competing interests

The authors declare that they have no competing interests.

## Authors contributions

YH analyzed the data, diagnosed and wrote the manuscript as a major contributor. XQ, QL, YH, XL, QZ, JW contributed to diagnose and management of the patient. EW carried out the histopathological evaluation and helped to write manuscript. ZL performed the operation. All authors have read and approved the final manuscript.
